# A SIMPLIFIED APPROACH FOR CLASSIFYING PHYSICAL RESILIENCE AMONG OLDER ADULTS: THE HEALTH ABC STUDY

**DOI:** 10.1093/geroni/igac059.1154

**Published:** 2022-12-20

**Authors:** Chenkai Wu, Tianze Lin

**Affiliations:** Duke Kunshan University, Kunshan, Jiangsu, China (People's Republic); Duke Kunshan University, Kunshan, Jiangsu, China (People's Republic)

## Abstract

Physical resilience is an emerging concept within the context of aging and geriatric medicine, and we previously developed and validated one such measure based on the mismatch between persons’ frailty level and comorbidity burden. We sought to develop a simplified version for classifying physical resilience. We also examined the agreement between the simplified version and the original approach and evaluated its predictive validity. We included 2,457 older adults from the Health, Aging, and Body Composition Study. We constructed a simplified version for quantifying physical resilience based on the comorbidity burden and level of frailty (score: 0-10). Participants were grouped by the number of diseases and classified into three groups—adapters, expected agers, and premature frailers—based on the mean and SD of frailty score (less than, within, or above one standard deviation of the mean). The Cohen’s kappa between the novel resilience classification and the original approach was 0.70, and the percentage of absolute agreement was 85.4%. We observed a steep increase in years of healthy and able life from premature frailers to adapters in the simplified resilience classifications. We developed a simplified version for quantifying physical resilience in a cohort of initially well-functioning older Black and White adults. The agreement between the simplified version and the original approach is high. Adapters had a longer healthy lifespan than expected agers and premature frailers. This user-friendly measure could help assess patients’ physical resilience in clinical settings.

